# The effect of a smaller spacer in the PASCAL Ace on residual mitral valve orifice area

**DOI:** 10.1007/s00392-023-02368-0

**Published:** 2024-01-25

**Authors:** Michael Paukovitsch, Dominik Felbel, Marijana Tadic, Mirjam Keßler, Jinny Scheffler, Matthias Gröger, Sinisa Markovic, Wolfgang Rottbauer, Leonhard Moritz Schneider

**Affiliations:** Ulm University Heart Center, Department of Cardiology, Albert-Einstein-Allee 23, 89081 Ulm, Germany

**Keywords:** Mitral valve orifice area, Mitral valve edge-to-edge repair, Iatrogenic stenosis

## Abstract

**Background:**

Mitral transcatheter edge-to-edge repair (M-TEER) is an established treatment for functional mitral regurgitation (FMR) associated with a risk of creating iatrogenic stenosis.

**Objectives:**

To investigate the impact of the P10 and its larger spacer compared to the narrower Ace and its smaller spacer on reduction of mitral valve orifice area (MVOA) during M-TEER.

**Methods:**

Consecutive patients undergoing M-TEER for treatment of severe FMR were screened retrospectively. Patients with a single PASCAL device implantation within the central segments of the MV leaflets, non-complex anatomy, and baseline MVOA ≥ 3.5cm^2^ were selected. Intraprocedural transesophageal echocardiography was used to compare MVOA reduction with 3D multiplanar reconstruction and direct planimetry. Device selection did not follow a prespecified MVOA threshold.

**Results:**

Seventy-two patients (81.0 years, IQR {74.3–85.0}) were included. In 32 patients, the P10 was implanted (44.4%). MR severity (*p* = 0.66), MR reduction (*p* = 0.73), and body surface area (*p* = 0.56) were comparable. Baseline MVOA tended to be smaller in P10 patients with the larger spacer (5.0 ± 1.1 vs. 5.4 ± 1.3cm^2^, *p* = 0.18), however, residual MVOA was larger in these patients (2.7 ± 0.7 vs. 2.3 ± 0.6cm^2^, *p* = 0.03). Accordingly, relative MVOA reduction was significantly less in P10 patients (− 45.9 ± 7.6 vs. − 56.3 ± 7.0%, *p* < 0.01). Indirect annuloplasty was more pronounced in Ace patients whereas mean transmitral gradients were similar.

**Conclusion:**

In FMR patients with non-complex anatomy, the larger spacer of the P10 maintains greater MVOA with similar MR reduction. Hence, the use of the PASCAL Ace device in patients with small MVOAs might correlate with a risk of both clinically relevant orifice reduction and even iatrogenic stenosis.

**Graphical Abstract:**

Seventy-two patients treated for functional mitral regurgitation (FMR) with the narrower PASCAL Ace featuring a smaller spacer (*n* = 40) or the broader P10 with a larger spacer (*n* = 32) were included in this study. Using 3D TEE and multiplanar reconstruction for direct planimetry, mitral valve orifice areas (MVOA) were measured before and after device implantation. Only patients with central device positioning were included. The dimensions of the PASCAL device platform are shown as well. Note the larger space and broader design of the P10 compared to the PASCAL Ace. The difference in MVOA reduction amounted to 10%, which translates into roughly 0.5 cm^2^ based on an average MV found in this study.

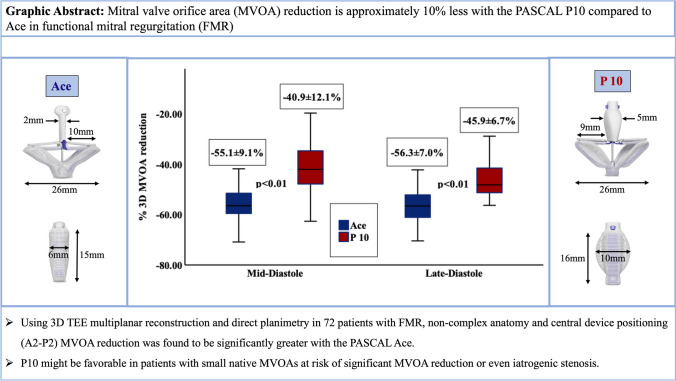

## Introduction

Transcatheter edge-to-edge repair (M-TEER) is an established treatment for degenerative (DMR) and functional (FMR) mitral regurgitation (MR). M-TEER is based on the principle of grasping and approximating the anterior and posterior mitral valve (MV) leaflets to reduce MR mimicking Alfieri’s stitch [1]. Similar to its surgical predecessor, M-TEER creates a double orifice [2] and reduces the overall MV orifice area (MVOA) [3, 4]. Previous studies mostly investigating Abbott’s MitraClip (Abbott Vascular, IL, USA) as the most experienced M-TEER system reported MVOA reduction between 30 and 65% in various populations including single and multiple device procedures [2, 5–12]. Accordingly, the recommended baseline preprocedural native MVOA for favorable M-TEER eligibility still is at least > 3.5cm^2^ and preferably larger [13], to avoid iatrogenic stenosis and adverse outcomes. However, especially in patients suffering from FMR, who remain symptomatic after guideline-directed medication and device therapy, if applicable, M-TEER often remains the only treatment option left even in smaller native MVOAs.

Apart from following the basic concept of M-TEER, the novel PASCAL system (Edwards Lifesciences) and its device platform introduced several unique design features such as the passive nitinol spring-based design and the concept of a central spacer [14]. The wider P10, the original PASCAL device, has paddles of 10mm width and 9mm insertion length clasping a 5 × 9 mm central spacer to fill in large central gaps, reduce leaflet stress, and further increase the residual MVOA at least in vitro. In contrast, the second-generation PASCAL Ace features a narrower design (6mm width, 10mm insertion length) and a smaller central spacer (2 × 5 mm). While a spacer may help fill the regurgitant orifice, the effects of the spacer and its different sizes in the PASCAL Ace and P10 on MVOA reduction in M-TEER are unknown. Especially, in vivo studies comparing MVOA reduction among the PASCAL platform are lacking. Thus, we retrospectively compared MVOA reduction with PASCAL P10 and Ace using 3D TEE data with multiplanar reconstruction (MPR) for direct planimetry in patients treated for FMR.

## Methods

### Study population and procedure

**Fig. 1:**
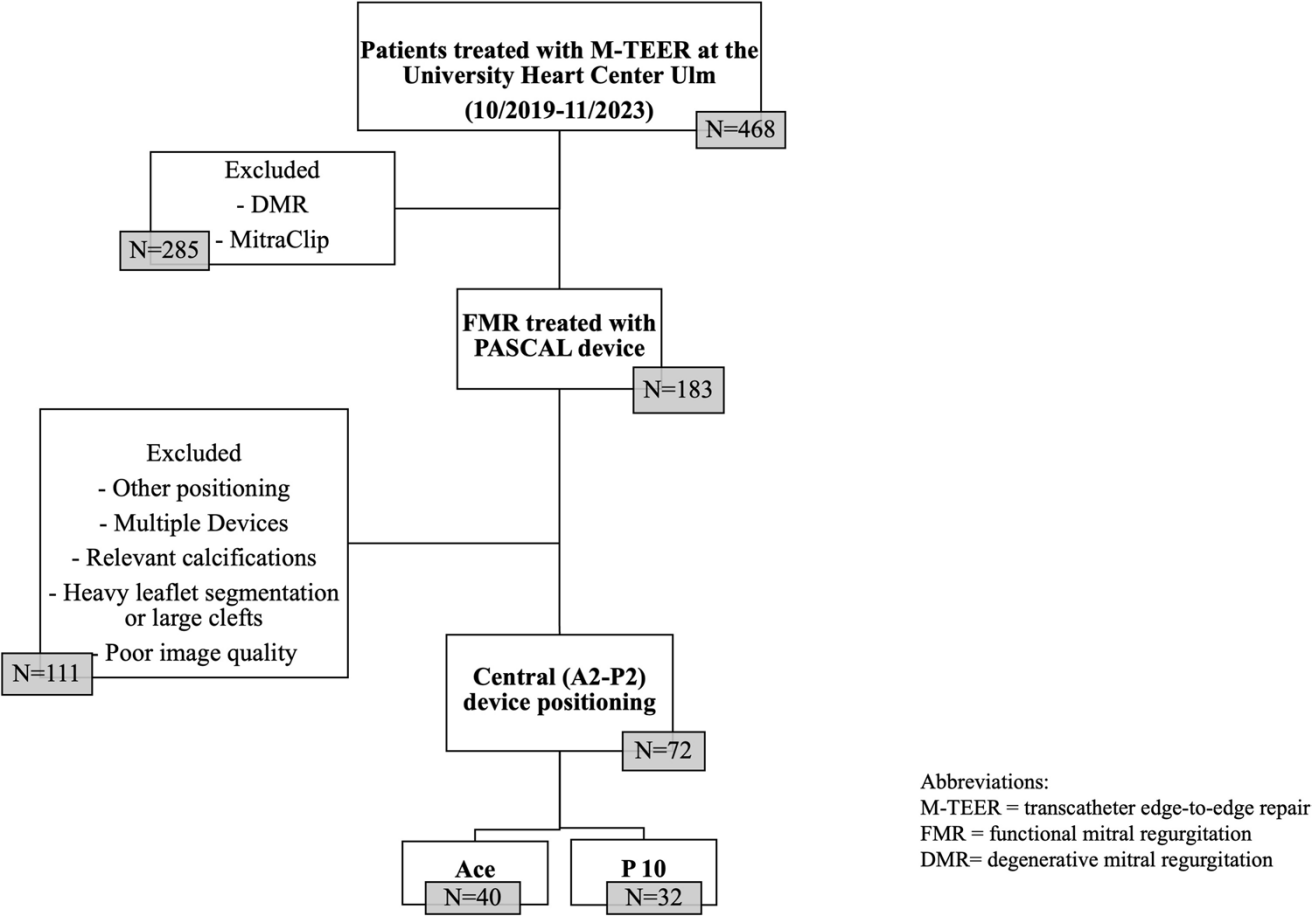
The study flowchart shows the inclusion and exclusion criteria for this study. After excluding patients with DMR, MitraClip implantation, multiple device implantation, and device positioning other than within the A2-P2 segments 72 patients remained for analysis

All patients treated with the PASCAL platform for symptomatic FMR grade III + /IV + at the University Heart Center Ulm between October 2019 and November 2023 were retrospectively evaluated for this investigation. Patients with a successful single device implantation (P10 or Ace) and central device positioning (A2-P2) were included. Exclusion criteria were multiple device implantation, device positioning other than A2-P2, MVOA ≤ 3.5 cm^2^, and poor image quality (see Fig. 1). To maximize comparability, further exclusion criteria were relevant calcifications, heavy leaflet segmentation or large clefts. Procedural images were acquired using a X8-t probe for transesophageal echochardiography (TEE) on an EPIQ™ ultrasound system (Philipps, Andover, MA, USA). Body surface area (BSA) was calculated using Mostseller’s formula [15]. All patients had been evaluated by the local heart team before referral to M-TEER treatment. All procedures were conducted under general anesthesia by our team of experienced interventional cardiologists. Device selection was within the operator’s discretion and did not follow a prespecified MVOA threshold. M-TEER is not recommended in patients with native MVOA ≤ 3.5 cm^2^ [13] and in general such patients do not receive M-TEER at our institution. Details of the procedure have been described before [16]. Briefly, after establishing femoral venous access and transseptal puncture to access the left atrium, either one of the PASCAL devices was implanted within the central MV guided by TEE and fluoroscopy. TEE guidance included all relevant intraprocedural measurements according to current guidelines such as MR reduction, pulmonary vein flow, MV gradients, orifice planimetry, pressure half-time (PHT), and stroke volume. Patients treated with either P10 or Ace were finally compared with regard to baseline characteristics and echocardiography, pre- and postprocedural MV geometry, and procedural results as well as pre- and postprocedural parameters related to MVOA reduction such as planimetric residual orifice area, MV gradients, and PHT.

This study was approved by the local ethics committee (Ethics Committee of Ulm University) and complies with the Declaration of Helsinki.

### MV measurements

**Fig. 2:**
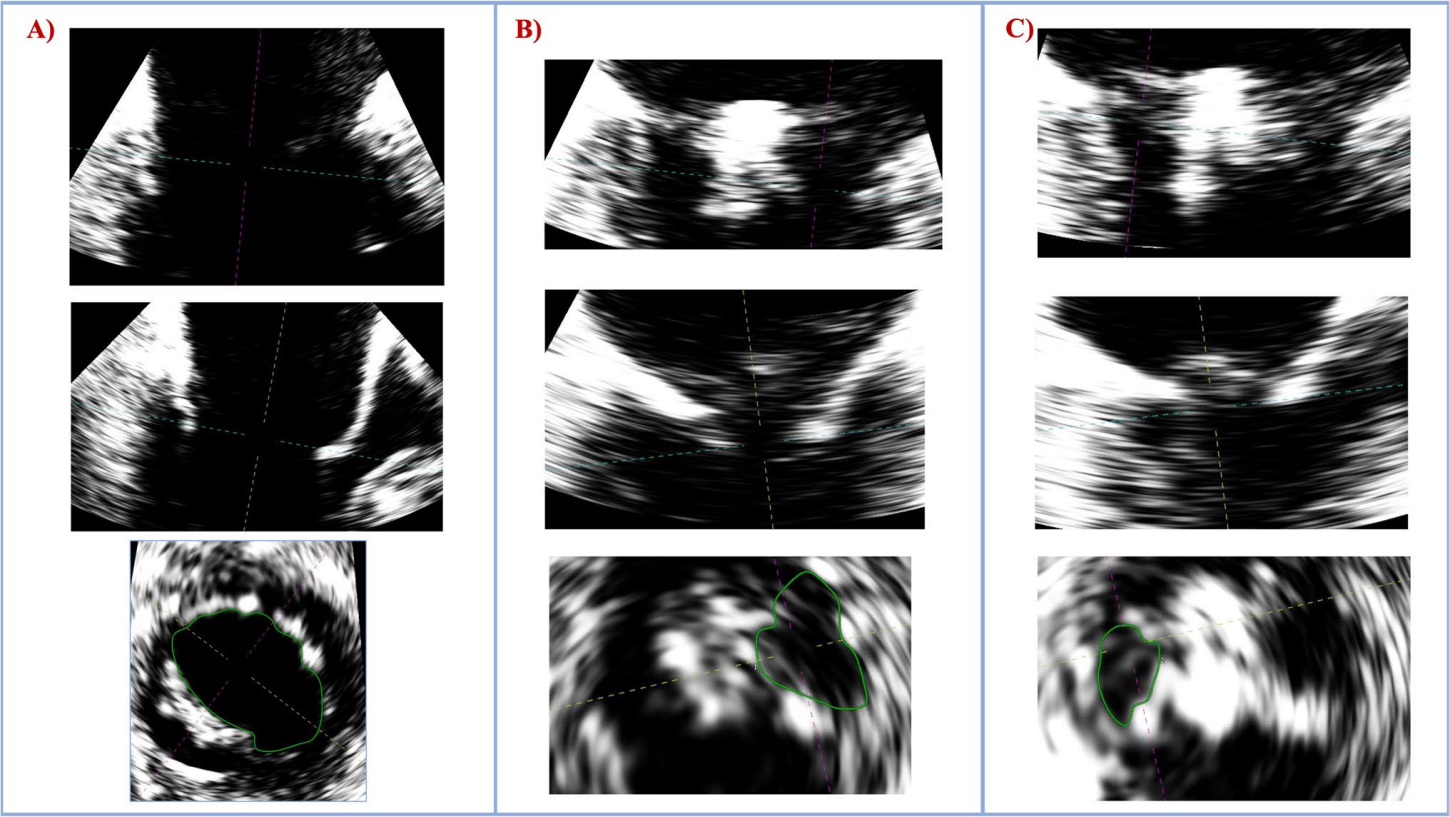
For correct planimetric MVOA measurement, the recorded loop is set at late diastole at maximum valve opening. The measuring plane is aligned at the leaflet tips both in preprocedural (**a**) and postprocedural measurement (**b** and **c**). In postprocedural assessment, the lateral (**b**) and medial orifice (**c**) are measured in a separate multiplanar reconstruction

Intraprocedural 3D TEE data sets of the MV were obtained from optimized mid-esophageal views and processed offline using a dedicated software (3D Cardio View, TOMTEC, Munich). Detailed assessment of pre- and postprocedural MV geometry has been described elsewhere [17]. MV geometry was assessed in end-diastole before and after device implantation. Leaflet length was measured during diastole at maximum elongation using straight distance or bend spacing where appropriate. MVOAs were quantified with direct planimetry at maximum valve opening in mid- as well as late diastole according to current guidelines and recommendations [18]: Using MPR, perpendicular planes were aligned at the leaflets’ tips in long-axis two-chamber and three-chamber views. Direct planimetry was then performed in the resulting short-axis view. After device implantation, double orifice measurements were performed likewise, however, separately for lateral and medial MVOAs, respectively. Figure 2 shows further details of the MVOA measurement before and after device implantation. In the case of patients with atrial fibrillation, MVOA measurement was performed selecting an RR interval of one beat occurring after two serial beats with an average RR interval, similar to a previous study [19]. Transmitral gradients were averaged over 5 cardiac cycles according to guidelines [20]. All measurements were performed by a single investigator (M. P.) and reviewed by a second investigator (L. S.).

### Statistical methods

Patients were analyzed in total and according to the PASCAL device used. The distribution of variables was analyzed graphically using histograms and Q-Q plots. Normally distributed variables are shown as mean ± standard deviation whereas non-normally distributed variables are shown as median and interquartile range (IQR). Groups were compared using the *T*-test and Mann–Whitney *U* test as appropriate. Categorical variables are shown as frequencies and percentages and were compared using the chi-square test. For paired variables, the paired *T*-test or Wilcoxon test was used as appropriate. All tests were performed two-sided and a *p*-value < 0.05 was considered significant. All testing was performed with SPSS, SPSS IBM, Version 29.

## Results

### Baseline characteristics

The study flowchart (Fig. 1) depicts the screening and selection process. From 468 patients treated with M-TEER during the inclusion period, a total of 72 FMR patients with a median age of 81.0 years (IQR {interquartile range}: 74.3–85.0 years) were deemed eligible for inclusion in this analysis (see also Fig. 1 and Table 1). In 40 (55.5%), the PASCAL Ace was implanted, whereas 32 (44.4%) patients received a P10 device.

**Table 1:** Baseline characteristics

	Total (*N* = 72)	Ace (*N* = 40)	P10 (*N* = 32)	*p*
Age, years	81.0 {74.3–85.0}	81.0 {77.0–87.9}	81.0 {73.3–83.8}	0.48
BMI, kg/m^2^	25.6 ± 4.7	25.3 ± 4.9	26.0 ± 4.4	0.53
BSA, m^2^	1.8 ± 0.3	1.8 ± 0.4	1.8 ± 0.2	0.56
Female, *N* (%)	35 (48.6)	18 (45.0)	17 (53.1)	0.64
aHT, *N* (%)	59 (81.9)	32 (80.0)	27 (84.4)	0.76
CAD, *N* (%)	48 (66.7)	29 (72.5)	19 (59.4)	0.32
Prior MI, *N* (%)	22 (30.6)	12 (30.0)	10 (31.3)	1.0
COPD, *N* (%)	9 (12.5)	4 (10.0)	5 (15.6)	0.5
Diabetes mellitus, *N* (%)	18 (25.0)	10 (25.0)	8 (25.0)	1.0
AFib, *N* (%)	50 (69.4)	26 (65.0)	24 (75.0)	0.44
CRT-D/P, *N* (%)	1 (2.0)	0	1 (3.1)	0.44
DCM, *N* (%)	18 (25.0)	7 (21.9)	11 (27.5)	0.78
NYHA III, *N* (%)	49 (68.1)	25 (62.5)	24 (75.0)	0.26
NYHA IV, *N* (%)	23 (31.9)	15 (37.5)	8 (25.0)	
Euro SCORE II, %	6.2 {3.5–11.2}	6.2 {3.8–10.9}	7.2 {3.3–11.4}	0.86
STS Score, %	5.0 {2.5–7.7}	5.3 {2.2–8.6}	4.7 {2.7–6.9}	0.81
Troponin T, µg/l	29.5 {18.3–55.0}	31.5 {17.3–60.5}	28.5 {19.3–43.0}	0.32
NT-proBNP, pg/ml	3200.0 {1102.0–10,562.0}	3719.0 {1424.3–10,727.0}	2901 {877.0–8407.0}	0.67
eGFR, ml/min	44.8 ± 20.0	44.1 ± 20.1	45.6 ± 19.4	0.75
CKD III/IV, *N* (%)	52 (72.2)	30 (75.0)	22 (68.8)	0.56
BB, N (%)	62 (86.1)	34 (85.0)	28 (87.5)	1.0
ACEI/ARB, *N* (%)	30 (41.7)	16 (40.0)	14 (43.8)	0.74
ARNI, *N* (%)	27 (37.5)	14 (35.0)	13 (40.6)	0.62
SGLT2-i, *N* (%)	33 (45.8)	19 (47.5)	14 (43.8)	0.84
MRA, *N* (%)	41 (56.9)	22 (55.0)	19 (59.4)	0.71

Values are shown as frequencies (*N*) and percentages (%), mean ± standard deviation (SD), or median and IQR in paranthesis

*ACEI* angiotensin-converting enzyme inhibitor, *AFib* atrial fibrillation, *aHT* arterial hypertension, *ARB* AT receptor blocker, *ARNI* angiotensin-neprilysin inhibitor, *BB* beta blocker, *BMI* body mass index (kg/m^2^), *BSA* body surface area, *CAD* coronary artery disease, *CKD* chronic kidney disease, *COPD* chronic obstructive pulmonary disease, *CRT* cardiac resynchronization therapy, *DCM* dilative cardiomyopathy, *eGFR* estimated glomerular filtration rate, *MRA* mineralocorticoid receptor antagonist, *NOAC* novel oral anticoagulant, *NT-proBNP* N-terminal prohormone brain natriuretic peptide, *NYHA* New York Heart Association, *MI* myocardial infarction, *SGLT2-i* sodium-glucose cotransporter-2 inhibitor, *STS* Society of Thoracic Surgeons

Bold numbers indicate significant *p*-values

Baseline characteristics were well balanced between both treatment groups (see also Table 1). Particularly, BSA (1.8 ± 0.4 vs. 1.8 ± 0.2 m^2^, *p* = 0.56) and body mass index (25.3 ± 4.9 vs. 26.0 ± 4.4 kg/m^2^, *p* = 0.53) were similar. Notably, also gender distribution did not differ between both groups resulting in 45.0 and 53.1% females in the Ace and P10 groups, respectively (*p* = 0.64). The burden of relevant comorbidities such as diabetes (*p* = 1.0), chronic renal disease (*p* = 0.56), or atrial fibrillation (*p* = 0.44) was also comparable between both cohorts.

### Baseline echocardiography and procedural results

Average left ventricular ejection fraction (LVEF) was reduced (41.1 ± 11.8%) in the overall cohort with similar LVEF in Ace and P10 patients (42.3 ± 12.4 vs. 39.7 ± 11.1%, *p* = 0.37; see also Table 2). Systolic pulmonary artery pressure (sPAP, *p* = 0.86) and tricuspid regurgitation (TR, *p* = 1.0) were similar as well. Moreover, there were no significant differences regarding MR severity before (*p* = 0.66 further see Table 2) and after device implantation (*p* = 0.73), which resulted in a similar rate of optimal (MR ≤ I) results in both groups (92.3 vs. 88.0%). Mean left atrial pressure before (*p* = 0.21) and after (*p* = 0.41) device implantation was found to be consistent and similar between both groups. Systolic blood pressure before (Ace: 115.0 {110.0–120} vs. 120.0 {106.3–123.8}, *p* = 0.03) and after (115.0 {106.0–120.0} vs. 120.0 {115.0–130.0}; *p* = 0.01) device implantation was significantly greater in P10 patients. There was no valid information on the respective utilization of catecholamines or volume substitution. The initial device intended to be used in a patient (either Ace or P10) was also the device finally implanted in all patients.

**Table 2:** Baseline echocardiography and procedural outcomes

	Total (*N* = 72)	Ace (*N* = 40)	P10 (*N* = 32)	*p*
LVEF, %	41.1 ± 11.8	42.3 ± 12.4	39.7 ± 11.1	0.37
LA diameter, mm	52.9 ± 7.9	54.3 ± 8.3	51.3 ± 7.3	0.2
sPAP, mmHg	48.6 ± 14.6	48.3 ± 14.5	49.0 ± 15.2	0.86
Severe TR, *N* (%)	18 (25.0)	10 (25.0)	8 (25.0)	1.0
Vena contracta, mm	7.0 ± 2.7	6.7 ± 3.1	7.4 ± 2.2	0.31
PISA radius, cm	0.7 ± 0.2	0.7 ± 0.1	0.7 ± 0.2	0.35
ERO A, cm^2^	0.22 ± 0.1	0.24 ± 0.1	0.21 ± 0.1	0.3
RV, ml	33.2 ± 15.8	34.4 ± 17.6	31.7 ± 13.4	0.5
Tenting height, mm	10.0 ± 4.0	9.0 ± 3.0	10.0 ± 4.0	0.32
AML, mm	32.7 ± 8.5	33.3 ± 10.0	32.0 ± 5.0	0.56
PML, mm	14.5 ± 6.1	14.5 ± 6.6	14.5 ± 5.6	0.99
Mean LAP pre, mmHg	18.0 {14.0–23.0}	18.0 {13.3–21.0}	20.0 {14.0–25.0}	0.21
Systolic BP pre, mmHg	115.0 {100.0–120.0}	115.0 {100–120.0}	120.0 {106.3–123.8}	**0.03**
Diastolic BP pre, mmHg	60.0 {50.0–65.0}	60.0 {50.0–65.0}	60.0 {50.0–65.0}	0.85
Grade of MR pre				
III, *N* (%)	47 (65.3)	27 (67.5)	20 (62.5)	0.66
IV, *N* (%)	25 (34.7)	13 (32.5)	12 (37.5)	
Procedure time (min)	60.0 (48.0–87.5)	56.5 (48.8–78.5)	63.0 (48.0–92.0)	0.46
Grade of MR post				
≤ I, *N* (%)	64 (88.9)	35 (87.5)	29 (90.6)	0.73
II, *N* (%)	8 (11.1)	5 (12.5)	3 (9.4)	
Mean LAP post, mmHg	17.0 {14.0–21.0}	16.0 {14.0–20.0}	19.0 {14.0–21.8}	0.32
Systolic BP post, mmHg	120.0 {110.0–120.0}	115.0 {106.0–120.0}	120.0 {115.0–130.0}	**0.01**
Diastolic BP post, mmHg	60.0 {58.8–70.0}	60.0 {50.0–70.0}	60.0 {60.0–70.0}	0.81

Values are shown as frequencies (*N*) and percentages (%) or mean ± standard deviation (SD) or median and IQR in paranthesis

*AML *anterior mitral leaflet*, BP* blood pressure, *ERO A* effective regurgitant orifice area, *LAP* left atrial pressure, *LVEF* left ventricular ejection fraction, *MR* mitral regurgitation, *PISA* proximal isovelocity surface area, *PML* posterior mitral leaflet, *RV* regurgitant volume, *sPAP* systolic pulmonary artery pressure, *TR* tricuspid regurgitation

Bold numbers indicate significant *p*-values

### MV measurements

Overall, there were no relevant differences in preprocedural MV geometry as well as in anterior (AML, *p* = 0.56) or posterior leaflet length (PML, *p* = 0.99) between patients treated with the PASCAL Ace or P10. Anterior–posterior (A-Pd) as well as anterolateral-posteromedial diameters (AL-PMd) and 2D and 3D annular area (AA) were similar in both patient cohorts before device implantation (see Table 3). Moreover, both groups showed a comparable tenting height (9.0 ± 3.0 vs. 10.0 ± 4.9 mm, *p* = 0.32).

**Table 3:** MVOA measurements, MV gradients, and geometrical assessment

	Total (*N* = 72)	Ace (*N* = 40)	P10 (*N* = 32)	*p*
Mid-diastolic measurement				
Planimetric MVOA pre, cm^2^	4.1 ± 1.0	4.2 ± 1.0	3.8 ± 0.9	0.12
Planimetric MVOA post, cm^2^	1.9 ± 0.7	1.7 ± 0.7	2.1 ± 0.8	**0.04**
* Lateral MVOA, cm*^*2*^	1.1 ± 0.4	1.0 ± 0.4	1.2 ± 0.4	**0.02**
* Medial MVOA, cm*^*2*^	0.9 ± 0.3	0.9 ± 0.3	1.0 ± 0.3	0.13
Relative reduction, %	− 48.7 ± 12.7	− 55.1 ± 9.1	− 40.9 ± 12.1	** < 0.01**
Maximum valve opening late diastole				
Planimetric MVOA pre, cm^2^	5.2 ± 1.2	5.4 ± 1.3	5.0 ± 1.1	0.18
Planimetric MVOA post, cm^2^	2.5 ± 0.7	2.3 ± 0.6	2.7 ± 0.7	**0.03**
* Lateral MVOA, cm*^*2*^	1.3 ± 0.5	1.2 ± 0.4	1.4 ± 0.6	**0.02**
* Medial MVOA, cm*^*2*^	1.2 ± 0.4	1.1 ± 0.4	1.3 ± 0.4	0.07
Relative reduction, %	− 51.7 ± 8.9	− 56.3 ± 7.0	− 45.9 ± 7.6	** < 0.01**
mPG pre, mmHg	1.5 ± 0.7	1.5 ± 0.6	1.6 ± 0.7	0.34
mPG post, mmHg	3.1 ± 1.2	3.1 ± 1.2	3.2 ± 1.0	0.6
PHT MVOA pre, cm^2^	4.3 ± 0.9	4.3 ± 0.9	4.1 ± 0.9	0.36
PHT MVOA post, cm^2^	2.5 ± 0.7	2.5 ± 0.7	2.4 ± 0.6	0.56
A-Pd pre, cm	3.8 ± 0.5	3.8 ± 0.4	3.8 ± 0.5	0.94
A-Pd post, cm	3.6 ± 0.4	3.5 ± 0.4	3.7 ± 0.5	**0.047**
Relative change, %	− 6.7 ± 4.7	− 9.2 ± 4.0	− 3.5 ± 3.4	** < 0.01**
AL-PMd pre, cm	4.1 ± 0.4	4.1 ± 0.4	4.1 ± 0.4	0.55
AL-PMd post, cm	4.0 ± 0.4	3.9 ± 0.4	4.1 ± 0.4	0.1
Relative change, %	− 1.7 ± 6.6	− 2.7 ± 6.6	− 0.5 ± 6.4	0.18
2D AA pre, cm^2^	12.2 ± 2.5	11.9 ± 2.2	12.6 ± 2.7	0.26
2D AA post, cm^2^	11.4 ± 2.5	10.8 ± 2.3	12.2 ± 2.5	**0.02**
Relative change, %	− 7.9 ± 13.9	− 12.1 ± 16.9	− 2.6 ± 5.7	** < 0.01**
3D AA pre, cm^2^	12.8 ± 2.5	12.5 ± 2.1	13.3 ± 2.8	0.2
3D AA post, cm^2^	12.0 ± 2.4	11.3 ± 2.2	12.8 ± 2.5	**0.01**
Relative change, %	− 6.7 ± 7.0	− 9.6 ± 6.7	− 3.1 ± 5.6	** < 0.01**

Abbreviations: *AA* annular area, *AL-PMd* anterolateral-posteromedial diameter, *A-Pd* anterior–posterior diameter, *MVOA* mitral valve orifice area, *mPG* mean transmitral pressure gradient, *MV* mitral valve, *PHT* pressure half time

Bold numbers indicate significant *p*-values

After device implantation, the relative reduction of A-Pd was found to be significantly greater in patients treated with the Ace device (− 9.2 ± 4.0 vs. − 3.5 ± 3.4%, *p* < 0.01). A similar effect was observed regarding the reduction of 2D (− 12.1 ± 16.9 vs. − 2.6 ± 5.7%, *p* < 0.01) and 3D AA (− 9.6 ± 6.7 vs. − 3.1 ± 5.6%, *p* < 0.01), which were also found to be greater in Ace patients.

### MVOA measurements and MV gradients

Table 3 shows MVOA measurements (also see Fig. 2) and MV gradients before and after device implantation. Baseline MVOA tended to be smaller in patients treated with the P10, although this finding was not statistically significant (mid-diastole: 4.2 ± 1.0 vs. 3.8 ± 0.9 cm^2^, *p* = 0.12; late diastole: 5.4 ± 1.3 vs. 5.0 ± 1.1 cm^2^, *p* = 0.18). Interestingly, the opposite was observed after device implantation, where the residual MVOA was significantly greater in P10 patients in mid- (1.7 ± 0.7 vs. 2.1 ± 0.8 cm^2^, *p* = 0.04) as well as late diastole (2.3 ± 0.6 vs. 2.7 ± 0.7 cm^2^, *p* = 0.03). Hence, relative MVOA reduction significantly differed between the treatment groups in mid- as well as late diastole at maximum valve opening (*p* < 0.01, respectively). The PASCAL Ace reduced the MVOA by 55.1 ± 9.1 and 56.3 ± 7.0%, whereas P10 implantation led to 40.9 ± 12.1 and 45.9 ± 7.6% relative MVOA reduction (see also Graphical abstract). Mean transmitral gradients (mPG) were similar before (1.5 ± 0.6 vs. 1.6 ± 0.7 mmHg, *p* = 0.34) and after device implantation (3.1 ± 1.2 vs. 3.2 ± 1.0, *p* = 0.6). Likewise, there was no relevant difference between pre- and postprocedural MVOAs measured by using the pressure half-time method (PHT) in patients treated with the Ace or PASCAL P10 devices (see Table 3).

## Discussion

M-TEER is an established treatment for MR that inherently reduces the MV opening and thereby bears a risk of creating iatrogenic stenosis. Accordingly, the therapeutic options are limited in smaller native MVOAs. On the other hand, additional clip sizes and the introduction of a second M-TEER system allow for increasingly differentiated valve repair even in complex MV anatomy. In contrast to the MitraClip, the PASCAL device platform features two sizes of a central spacer, intended to tackle central MR jets [14]. Furthermore, the size of the spacer might influence leaflet mobility, indirect MV annuloplasty, and residual MVOA. Apart from that, the overall smaller design of the Ace seems to reduce echo shading, a feature especially useful in tricuspid edge-to-edge repair.

In this investigation, the effects of spacer size on planimetric residual MVOA were compared between the wider PASCAL P10 with the bigger spacer and the narrower Ace with the smaller spacer.

We included 72 FMR patients with central device positioning and were able to provide novel in vivo evidence of approximately 10% greater MVOA reduction with the Ace compared to the PASCAL P10 despite its almost twofold width.

### Echocardiographic assessment and comparability

MVOA measurement can be technically challenging and the exact positioning of the measurement plane at the leaflet tips is a prerequisite for adequate results. Accordingly, there is a risk of underestimating MVOA reduction when measurement is performed above the leaflet tips [8], and even subtle differences in measurement technique, such as angulations of the echo probe, may influence results and complicate the comparability of studies. Apart from that, valve-specific anatomical variations may impact M-TEER-induced MVOA reduction. However, using 3D TEE and MPR for direct planimetry previously showed superior accuracy in predicting postprocedural stenosis compared to TTE and 2D TEE MVOA [21] and represents the recommended standard for intraprocedural imaging [18].

Current evidence further indicates that MVOA reduction differs among the available devices and strongly depends on device positioning. Kassar et al. [10] recently reported significantly greater MVOA reduction by using the MitraClip XTR compared to NTR. Moreover, central or paracentral device positioning was associated with greater MVOA reduction compared to commissural positioning [10]. All MVOA measurements in this investigation were performed offline using dedicated software and a detailed protocol. Moreover, only single device procedures and central positioning in non-complex MV anatomy were included to maximize comparability.

### M-TEER-induced MVOA reduction

Previous studies reporting on MVOA reduction mostly focused on the MitraClip device [2, 5–12]. In these investigations, MVOA reduction ranged from 30.4 to 65.2% [2, 5–12], whereas studies exclusively including FMR patients reported MVOA reduction between 52.2 and 65.2% [6, 8, 9]. Since the introduction of the PASCAL platform, several studies were able to confirm feasibility and effectiveness [22] as well as similar MR reduction compared to the MitraClip [23]. Data on MVOA reduction using this novel system and its unique design features, however, is scarce. In 2017, Praz et al. [14] reported the first-in-man compassionate use of the PASCAL system and the P10 device in a multicenter, observational trial including 23 patients suffering from either DMR or FMR. MVOA reduction was found to be 47% on average with a single device implantation [14]. A recent study directly compared the MitraClip and PASCAL platforms in a cohort of 100 patients with single as well as multiple device procedures and showed significant MVOA reduction with both M-TEER systems [24]. Rosch et al. [24] included 50 patients mostly treated with the PASCAL P10 (94%) and 50 patients treated with the MitraClip XTR and/or NTR and compared MVOA reduction at five different time points during diastole and not only at maximum valve opening as previously applied in other studies [3, 5–7, 19, 25]. Interestingly, the MitraClip cohort showed greater MVOA reduction than patients treated with PASCAL [24] and the authors suggested that Edwards’ flexible nitinol design might preserve leaflet motion better compared to Abbott’s more rigid MitraClip [24]. MVOA reduction ranged roughly between 30 and 48% at various measurement points during diastole [24], which is in line with our findings (40.9 and 45.9%) and supports the potential role of a central spacer in preserving valve opening. Eventually, we observed an approximately 10% additional MVOA reduction by using the PASCAL Ace (55.1 and 56.3%) despite its smaller dimensions. Absolute values showed a non-significant trend toward smaller baseline MVOA in patients treated with the P10 and yet significantly greater residual MVOA was found in P10 patients. Notably, previous studies also focused on relative MVOA reduction [5, 6, 10]. Furthermore, we were able to show the association between spacer size, MVOA reduction, and indirect annuloplasty with greater A-Pd reduction in PASCAL Ace patients. A relevant finding, as the Ace was added to the PASCAL portfolio initially to extend therapeutic options, specifically in treating DMR where maximum leaflet insertion and tissue shortening are desired. A similar comparison of residual MVOA and MV geometry between PASCAL P10 and Ace in DMR should be the subject of future investigations to further explore device-specific advantages and lead to patient-tailored therapies.

### Residual MVOA and MV gradients

Even though iatrogenic stenosis after M-TEER is not a frequent complication, it has been associated with adverse outcomes [21, 26]. The most commonly used parameter to assess M-TEER-induced stenosis still is mPG. However, according to criteria laid out by the Mitral Valve Academic Research Council, either mPG exceeding 5mmHg or residual MVOA ≤ 1.5cm^2^ is considered device failure [27]. Transmitral gradients depend on volume load, cardiac mechanics, and heart rate unlike direct MVOA planimetry. Notably, the significant difference in relative planimetric MVOA reduction between PASCAL P10 and Ace observed in our study did not translate into a relevant difference in pre- and postprocedural mPG. Since echocardiographic mPG are highly variable, direct planimetry may be a superior approach. A recent investigation by Hadjadj et al. [21] reported that 3D MVOA indexed to body surface area and 3D MVOA indexed to stroke volume could be potential predictors of M-TEER-induced mitral stenosis. By all means, the hemodynamic impact of a respective MVOA reduction and its clinical implication should be assessed by multiple echocardiographic parameters, which should in turn be put in relation to patient specifics and hemodynamic response.

However, given the average preprocedural MVOA of 5.2 cm^2^ in the overall study population, neither one of the PASCAL devices was even close to creating stenosis. Eventually, smaller preprocedural MVOAs have repeatedly been associated with iatrogenic stenosis after M-TEER [6, 21, 26] and according to this, a difference in MV gradients between PASCAL P10 and Ace might only be observable in a population with preprocedural MVOAs < 4cm^2^. The impact of a 10% difference in relative MVOA reduction on mPG might become more evident in such a population likewise. Eventually, mean left atrial pressure did not decrease considerably after M-TEER in either device group, which is a frequent finding in FMR. However, missing information on circulatory support during anesthesia limits any conclusion regarding implications on hemodynamics in this investigation.

## Limitations

We present results from a retrospective single-center study with all its inherent limitations. Although device selection was not triggered by any MVOA threshold and baseline characteristics as well as valve-specific findings were comparable, other factors which might impact our results can not be ultimately ruled out. We investigated FMR patients with a single PASCAL device implantation, non-complex anatomy, and central device positioning (A2-P2) exclusively, which limited inclusion and led to a relatively small sample size. Any result can therefore not be translated to patients with DMR or non-central device positioning. Measurements were performed by a single investigator (M. P.) and reviewed by a second (L. S.) investigator, which were not blinded by the device used during the respective procedure. Finally, all results must be considered hypothesis-generating in terms of avoiding iatrogenic stenosis in smaller native MVOA and warrant further confirmation.

## Conclusion

In this retrospective single-center study, M-TEER using the P10 compared to the PASCAL Ace in non-complex FMR leads to approximately 10% less MVOA reduction despite its almost twofold width. Use of the PASCAL Ace in patients with small native MVOAs might carry a risk of creating clinically relevant MVOA reduction and iatrogenic stenosis.

As these findings might impact the differential use of these devices, a randomized-controlled trial is warranted to ultimately confirm this effect.

## Abbreviations

A-Pd: Anterior-posterior diameter
AA: Mitral annular area
FMR: Functional mitral regurgitation
DMR: Degenerative mitral regurgitation
M-TEER: Mitral transcatheter edge-to-edge repair
MPR: Multiplanar reconstruction
MR: Mitral regurgitation
MV: Mitral valve
MVOA: Mitral valve orifice area
TEE: Transesophageal echocardiography
